# *Notes from the Field:* Early Evidence of the SARS-CoV-2 B.1.1.529 (Omicron) Variant in Community Wastewater — United States, November–December 2021

**DOI:** 10.15585/mmwr.mm7103a5

**Published:** 2022-01-21

**Authors:** Amy E. Kirby, Rory M. Welsh, Zachary A. Marsh, Alexander T. Yu, Duc J. Vugia, Alexandria B. Boehm, Marlene K. Wolfe, Bradley J. White, Shannon R. Matzinger, Allison Wheeler, Laura Bankers, Kevin Andresen, Cristal Salatas, New York City Department of Environmental Protection, Devon A. Gregory, Marc C. Johnson, Monica Trujillo, Sherin Kannoly, Davida S. Smyth, John J. Dennehy, Nicolae Sapoval, Katherine Ensor, Todd Treangen, Lauren B. Stadler, Loren Hopkins

**Affiliations:** ^1^Division of Foodborne, Waterborne, and Environmental Diseases, National Center for Emerging and Zoonotic Infectious Diseases, CDC; ^2^California Department of Public Health; ^3^Stanford University, Stanford, California; ^4^Rollins School of Public Health, Emory University, Atlanta, Georgia; ^5^Verily Life Sciences, South San Francisco, California; ^6^Colorado Department of Public Health and Environment; ^7^University of Missouri-School of Medicine, Columbia, Missouri; ^8^Queens College, The City University of New York, New York; ^9^Biology Doctoral Program, The Graduate Center, The City University of New York, New York;^ 10^Queensborough Community College, The City University of New York, New York; ^11^Texas A&M University-San Antonio, San Antonio, Texas; ^12^Rice University, Houston, Texas; ^13^Houston Health Department, Houston, Texas.

The United States designated the B.1.1.529 (Omicron) variant of SARS-CoV-2 (the virus that causes COVID-19) a variant of concern on November 30, 2021, and the first U.S. Omicron COVID-19 case was reported on December 1 (*1*). By December 18, Omicron was estimated to account for 37.9% of U.S. COVID-19 cases.* Early warning systems, such as sewage (wastewater) surveillance,^†^ can help track the spread of SARS-CoV-2 variants across communities (*2*).

The National Wastewater Surveillance System (NWSS) comprises 43 health departments funded by CDC to provide data on presence of and trends in SARS-CoV-2 infections that are independent of clinical testing. In addition to total SARS-CoV-2 testing, some health departments track SARS-CoV-2 variants by detecting variant-associated mutations in wastewater. Health departments in four states (California, Colorado, New York, and Texas) were the first wastewater surveillance programs to detect evidence of Omicron in community wastewater. This report describes the initial detections in wastewater during November 21–December 16, 2021, and the interpretative framework for these types of data. This activity was reviewed by CDC and was conducted consistent with applicable federal law and CDC policy.^§^

## California

The California Department of Public Health and academic partners use mutation-specific reverse transcription–polymerase chain reaction (RT-PCR) and sequencing to track variants in wastewater collected daily from 10 sewersheds.^¶^^,^** Omicron-associated mutations delHV69–70 (also seen with Alpha variant [B.1.1.7 and Q lineages])^††^ and del143–145 were detected in samples collected November 25 and November 30, 2021, from two Northern California communities (Table). Results from these samples were available on December 2; at that time, two clinical COVID-19 cases attributed to Omicron had been identified in California, but none from these communities. By December 17, del143–145 mutations were detected at all 10 sampled sewersheds in California communities.

**TABLE:** Detection of mutations associated with the SARS-CoV-2 B.1.1.529 (Omicron) variant in wastewater — California, Colorado, New York City, and Houston, Texas, November 21–December 16, 2021

Location	Sample date	Test method	Results
**California**			
Sewershed A	Nov 25, 2021	Mutation-specific RT-PCRs targeting delHV69–70 and del143–145*	Both mutations detected at <1,000 genomic copies/gram wastewater solids
Sewershed B	Nov 30, 2021	Mutation-specific RT-PCRs targeting delHV69–70 and del143–145*	Both mutations detected at <1,000 genomic copies/gram wastewater solids
	Dec 2, 2021	Mutation-specific RT-PCRs targeting delHV69–70 and del143–145*	Both mutations detected at <1,000 genomic copies/gram wastewater solids
		Partial sequencing of S-gene using ARTIC v4 73R, 74L primers	Detected 9 bp insertion mutation in s214EPE and 3 bp N211I deletion
Sewersheds (10 sites)	Dec 17, 2021	Mutation-specific RT-PCR targeting del143–145*	Mutations detected at >4,500 genomic copies/gram wastewater solids
	10 of 10 sites		
**Colorado**			
Sewersheds (21 sites)	Dec 2, 2021	SARS-CoV-2-enriched tiled amplicon sequencing	Detected 13 of 17 Omicron-associated mutations
	One of 21 sites		
	Dec 6, 2021	SARS-CoV-2-enriched tiled amplicon sequencing	No Omicron-associated mutations detected
	Zero of 21 sites		
	Dec 9, 2021	SARS-CoV-2-enriched tiled amplicon sequencing	Detected between four and 13 of 17 Omicron-associated mutations depending on the site
	Five of 21 sites		
	Dec 13, 2021	SARS-CoV-2-enriched tiled amplicon sequencing	Detected between six and 14 of 17 Omicron-associated mutations, depending on the site
	12 of 21 sites		
	Dec 16, 2021	SARS-CoV-2-enriched tiled amplicon sequencing	Detected between 12 and 14 of 17 Omicron-associated mutations, depending on the site
	19 of 21 sites		
**New York City**			
Sewershed A	Nov 21, 2021	Short-read sequencing of S-gene amplicon^†,§^	Detected 12 Omicron-associated mutations including eight mutations unique to Omicron
	Nov 28, 2021	Short-read sequencing of S-gene amplicon^†,§^	Detected 12 Omicron-associated mutations including eight mutations unique to Omicron
Sewershed B	Nov 28, 2021	Short-read sequencing of S-gene amplicon^†,§^	Detected 12 Omicron-associated mutations including eight mutations unique to Omicron
**Houston, Texas**			
Sewersheds (39 sites)	Nov 29, 2021	SARS-CoV-2-enriched tiled amplicon sequencing using ARTIC v3 primers^¶^	Detected six Omicron-associated mutations
	Seven of 39 sites		
	Dec 6, 2021	SARS-CoV-2-enriched tiled amplicon sequencing using ARTIC v3 primers^¶^	Detected 14 Omicron-associated mutations
	25 of 39 sites		
	Dec 13, 2021	SARS-CoV-2-enriched tiled amplicon sequencing using ARTIC v3 primers^¶^	Detected 18 Omicron-associated mutations
	35 of 39 sites		

**Abbreviation:** RT-PCR = reverse transcription–polymerase chain reaction.

* https://www.protocols.io/view/quantification-of-sars-cov-2-variant-mutations-hv6-b2qmqdu6

^†^ https://www.medrxiv.org/content/10.1101/2021.03.21.21253978v1

^§^ https://www.medrxiv.org/content/10.1101/2021.07.26.21261142v1

^¶^ https://www.medrxiv.org/content/10.1101/2021.09.08.21263279v1

## Colorado

The Colorado Department of Public Health and Environment conducts biweekly SARS-CoV-2 wastewater testing at 21 sewersheds,^§§^ using sequencing to track variants. Thirteen Omicron-associated mutations were detected in a sample collected on December 2, 2021. At that time, only one travel-associated Omicron case had been reported in Colorado. No Omicron-associated mutations were detected in the samples collected on December 6; however, by December 16, Omicron-associated mutations were detected at 19 of 21 sewersheds.

## New York City

The New York City Department of Environmental Protection tracks variants in wastewater by sequencing weekly samples collected from 14 sewersheds^¶¶^^,^*** (*3*). Twelve Omicron-associated mutations were detected in a sample collected on November 21. By December 4, the date the wastewater data were reported, one Omicron case had been identified in a resident of the sewershed. Samples collected on November 28 from this same sewershed and from another sewershed contained Omicron-associated mutations, as reported to the health department on December 17.

## Houston, Texas

The Houston Health Department conducts weekly wastewater testing at 39 sewersheds in the city and uses sequencing to track variants.^†††^ Sequencing detected six Omicron-associated mutations in samples collected on November 29 from seven sewersheds across the city. The first clinical detection of Omicron in the city was reported on December 1. The number of Omicron-positive sites, as well as the number of Omicron-associated mutations detected, increased over the subsequent 2 weeks.

## Discussion

The wastewater surveillance programs in these four states were the first to detect evidence of Omicron in community wastewater. Variant tracking data from wastewater cannot confirm the presence of a specific variant because the methods used cannot determine whether all variant-defining mutations are present on a single genome. However, conditions that increase confidence in the results include detection of multiple variant-associated mutations; linked mutations (i.e., on the same sequence read), or unique mutations not shared by other known variants; RNA concentration data consistent with emergence (e.g., low initial concentrations, increasing over time); the reporting of clinical cases in the area; detections in consecutive samples or via multiple methods; and RNA concentration or sequence abundance data for multiple variant-associated mutations trending together. Limitations of variant tracking in wastewater include detections inconsistent with the current epidemiology, low quality sequence data, sporadic detections, detection of a single variant-associated mutation, and conflicting trends in concentration or abundance data for mutations associated with the same variant. Reporting times >1 week can limit the usefulness of this data.

The detection of Omicron-associated mutations in community wastewater provides strong early evidence that the Omicron variant was likely present or more widely distributed in these communities than originally indicated by clinical testing alone; Omicron-associated mutations were documented during November 2021, at least a week before the first U.S. case identified via clinical testing on December 1. Variant tracking data from wastewater can be used as a complement to clinical testing for early detection of emerging variants, which can help guide decisions about allocation of clinical and public health resources, testing strategies, and public health messaging.

## Footnotes

* https://covid.cdc.gov/covid-data-tracker/#variant-proportions (Accessed January 10, 2022).

†
https://www.cdc.gov/healthywater/surveillance/wastewater-surveillance/wastewater-surveillance.html

§ 45 C.F.R. part 46, 21 C.F.R. part 56; 42 U.S.C. Sect. 241(d); 5 U.S.C. Sect. 552a; 44 U.S.C. Sect. 3501 et seq.

¶ Quantitative SARS-CoV-2 measurements in untreated sewage can provide information on changes in total SARS-CoV-2 infection in the community contributing to that wastewater treatment plant. That area is known as the sewershed.

**
https://www.protocols.io/view/quantification-of-sars-cov-2-variant-mutations-hv6-b2qmqdu6

††
https://www.researchsquare.com/article/rs-1083575/v1

§§
https://covid19.colorado.gov/covid-19-monitoring-in-wastewater

¶¶
https://www.medrxiv.org/content/10.1101/2021.03.21.21253978v1

***
https://www.medrxiv.org/content/10.1101/2021.07.26.21261142v1

†††
https://www.medrxiv.org/content/10.1101/2021.09.08.21263279v1
